# Numerical Studies on Damage Behavior of Recycled Aggregate Concrete Based on a 3D Model

**DOI:** 10.3390/ma13020355

**Published:** 2020-01-12

**Authors:** Yao Wang, Huawei Zhao, Minyao Xu, Chunyang Wu, Jiajia Fu, Lili Gao, Mahmoud M. A. Kamel

**Affiliations:** 1 Department of Architecture and Engineering, Yancheng Polytechnic College, Jiangsu 224005, China; miaoyun@njtech.edu.cn (M.X.); zhhpan@just.edu.cn (C.W.); njtfjj@njtech.edu.cn (J.F.); weimin@khu.ac.kr (L.G.); 2 Department of Architecture and Engineering, Beijing University of Technology, Beijing 100124, China; mahmoud.kamel@fagoum.edu.eg; 3 School of Civil Engineering, Nanjing Technology University, Nanjing 211816, China; 4 Department of Civil Engineering, Faculty of Engineering, Fayoum University, Fayoum 63514, Egypt

**Keywords:** 3D BFEM, recycled aggregate concrete, numerical simulation, failure pattern

## Abstract

This paper develops a 3D base force element method (BFEM) based on the potential energy principle. According to the BFEM, the stiffness matrix and node displacement of any eight-node hexahedral element are derived as a uniform expression. Moreover, this expression is explicitly expressed without a Gaussian integral. A 3D random numerical model of recycled aggregate concrete (RAC) is established. The randomness of aggregate was obtained by using the Monte Carlo random method. The effects of the recycled aggregate substitution and adhered mortar percentage on the elastic modulus and compressive strength are explored under uniaxial compression loading. In addition, the failure pattern is also studied. The obtained data show that the 3D BFEM is an efficient method to explore the failure mechanism of heterogeneous materials. The 3D random RAC model is feasible for characterizing the mesostructure of RAC. Both the substitution of recycled aggregate and the percentage of adhering mortar have a non-negligible influence on the mechanical properties of RAC. As the weak points in the specimen, the old interfacial transition zone (ITZ) and adhered mortar are the major factors that lead to the weakened properties of RAC. The first crack always appears in these weak zones, and then, due to the increase and transfer of stress, approximately two-to-three continuous cracks are formed in the 45°direction of the specimen.

## 1. Introduction 

In the last several years, recycled aggregate concrete (RAC) has become a popularly used construction material that can effectively alleviate the shortage of natural resources. As a kind of green building material, RAC has attracted many researchers to explore its mechanical performance [1,2,3,4,5]. Compared to natural aggregate concrete (NAC), RAC has a highly heterogeneous internal composite, and its mechanical behavior is related to the mesostructure of its components. The literature has revealed that the substitution of recycled aggregate, water/cement ratio, aggregate content, the percentage of adhered mortar, air content, etc. play a significant role in the mechanical properties of RAC [6,7]. At the mesostructural level, the component of RAC identified to be a five-phase system including recycled aggregate, adhered mortar, new cement mortar, an old interfacial transition zone (ITZ), and a new ITZ. There are two ways to explore the mechanical properties of engineering materials, namely macroscopic experimental tests and mesoscopic numerical simulations. Since the concept of the finite element method (FEM) was proposed by Clough in the 1960s [8], FEM has become an effective and accurate approach for assisting macroscopic experiments [9,10,11,12,13]. In addition, this method reduces the consumption of natural resources, time, and testing costs.

At the mesoscopic level, lots of work about the modelling of aggregate have been conducted by many researchers. For the simulation of concrete, a numerical concrete concept was proposed by Zaitsev and Wittmann, and three random 2D structures including spherical geometry, polygons and arbitrary polygonal were first generated based on meso-mechanics [14,15]. Subsequently, different mesoscale structures were proposed to simulate the concrete fracture process according to the FEM. For instance, Peng et al. [16] established a model of a circular aggregate model to explore the mechanical properties of concrete according to the Walraven formula [17] and the Monte Carlo random sampling principle. Additionally, based on the Monte Carlo random sampling principle, a particle model was established to represent the fragile aggregates by Bazant et al. [18]. Wang et al. [7] proposed a convex aggregate to model crushed stone based on a round aggregate. Wriggers et al. [19] and Chen et al. [20] proposed a 3D geometrical model for NAC according to the random mesostructure of natural aggregates in a specimen.

These natural aggregate models have provided effective reference methods to simulate recycled aggregate. Some researchers conducted a series of numerical research studies, and some conclusions have been obtained about RAC. Xiao et al. [11] designed a nine recycled aggregate model to study the effect of the relative elastic modulus of ITZs of cement mortar on the damage crack of RAC under uniaxial compression and tensile loading; the obtained data showed that the relative elastic modulus had a major effect on the stress–strain curves and RAC strength. Sun et al. [21] presented a 3D FEM model to research the effect of recycled aggregate substitution on shear strength by using the ABAQUS/Standard module software. The data showed that the shear stress was reduced by up to 13.8% by ranging the substitution from 0% to 100%. Chen et al. [22] designed four levels of recycled aggregate substitution to explore the damage mechanism of RAC under uniaxial compression loading. The data showed that the compressive strength reduced as the replacement ratio increased. Jayasuriya et al. [23] presented four different adhered mortar percentages to analyze the effect of adhering mortar on compressive strength, and their numerical data showed that the compressive strength was reduced up to 9% as changing the adhering mortar from 2% to 50%. Wang et al. [7] established two types of aggregate shapes to explore the effect of recycled aggregate replacement ratios on mechanical properties, and their simulation results demonstrated that the elastic modulus reduced by up to 16%~25%, and the compressive strength reduced by up to 12%~15% as the replacement ratios increased from 0% to 100%. Due to the exceedingly complicated stiffness matrix and multiple degrees of freedom per each element in the 3D level, a structure and mesh topology was rarely generated [19]. Most numerical simulations of RAC mechanical properties have been concentrated on the 2D level and scaled up by a thickness of the fictitious slice, and only a little consideration has been focused on 3D models of RAC.

As mentioned previously, the FEM has been proven to be an efficient approach to explore the mechanical properties of materials. In recent years, according to the potential energy principle, a new FEM concept and a new 2D FEM method were presented by Gao [24] and Peng et al. [25], named base force and the base force element method (BFEM), respectively. Based on the BFEM, the element stiffness matrix is conveyed by an explicit tensor formulation for an element with an arbitrary shape in any coordinate system. Moreover, the Gaussian integrals are not used in the calculations and deriving processes of the element stiffness matrix. 

For this paper, the 2D BFEM was developed into a 3D BFEM. In addition, a hexahedron element was established. A 3D numerical RAC model was established according to the 3D Fuller grading curve and the Monte Carlo random sampling method. The recycled aggregate was assumed to be a spherical particle. Several numerical models of RAC with five substitutions of recycled aggregate and six different percentages of adhered mortar were designed. These 3D RAC models were subjected to uniaxial compression loading that was controlled by displacement. The effects of the recycled aggregate replacement ratio and the adhered mortar content on the compressive strength and elastic modulus were investigated. Additionally, the failure pattern of a 3D RAC model was also calculated. 

## 2. Basic Formula of 3D Base Force Theory

For a 3D region of a solid medium in the Lagrangian coordinate system, $x^{i}\left(i=1,2,3\right)$ denotes the coordinate axes, and P,Q denote the initial/after position vector. The base vectors of material points can be defined as:

(1) $$P_{i}=\frac{\partial P}{\partial x^{i}},Q_{i}=\frac{\partial Q}{\partial x^{i}}$$

The displacement gradient can be expressed by the base vectors, as follows:

(2) $$u_{i}=\frac{\partial u}{\partial x^{i}}=P_{i}-Q_{i}=\frac{\partial P}{\partial x^{i}}-\frac{\partial Q}{\partial x^{i}}$$

**Figure 1:**
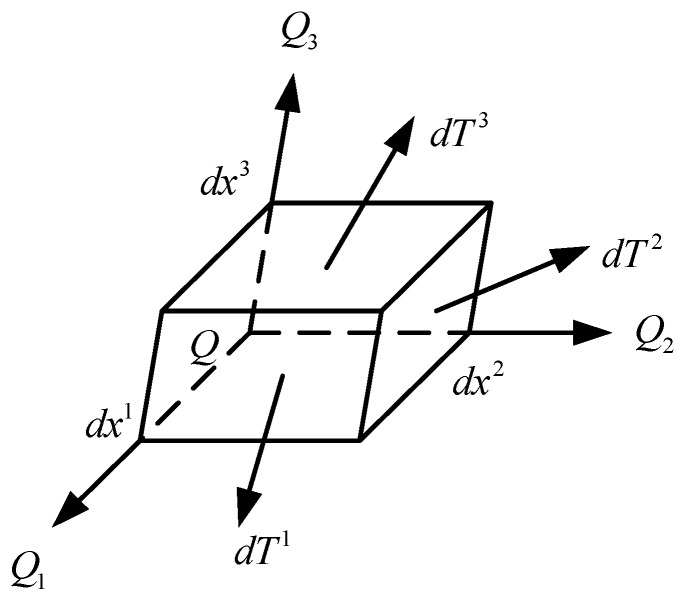
Base forces.

To express the stress state of the point Q, assuming a current configuration of this solid medium in the Cartesian coordinate system $x^{i}$, define a parallel hexahedron element and let $dx^{1},dx^{2},dx^{3}$ denote the element edges, as illustrated in Figure 1.

The force applied on the front surface of the element is marked as $dT^{i}$, so let:

(3) $$T^{i}=\frac{1}{dx^{i+1}dx^{i-1}}dT^{i}dx^{i}\to 0$$

In Equation (3), the indexes are promised 3 + 1 = 1 and 1 − 1 = 3, where $T^{i}\left(i=1,2,3\right)$ is the base force acting on the point Q in the 3D coordinate system $x^{i}$.

**Figure 2:**
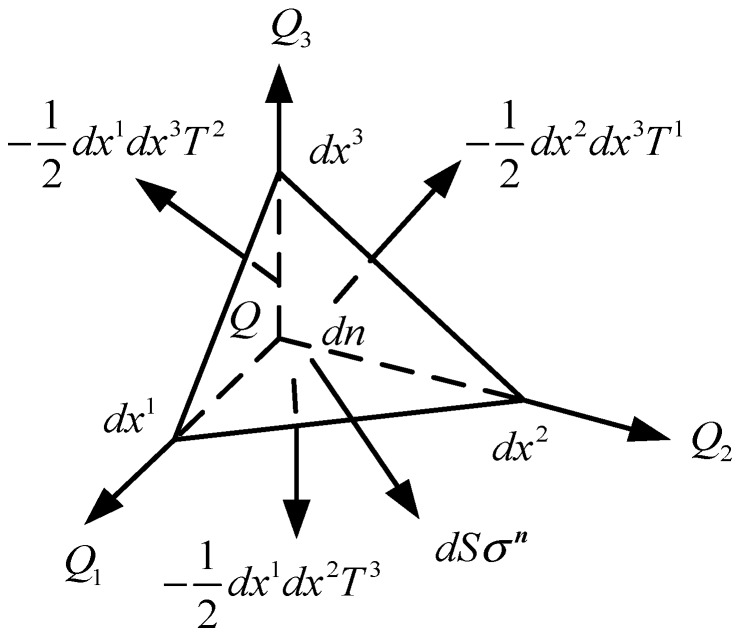
Forces on a tetrahedron.

To describe $T^{i}\left(i=1,2,3\right)$, define an arbitrary plane with normal n. The plane and coordinate axes $x^{i}$ intersect at $dx^{1},dx^{2},dx^{3}$, as shown in Figure 2. According to the equilibrium condition, the stress vector that acts on the surface can be obtained as:

(4) $$\sigma^{n}dS=\frac{1}{2}dx^{1}dx^{2}dx^{3}\left(\frac{1}{dx^{1}}T^{1}+\frac{1}{dx^{2}}T^{2}+\frac{1}{dx^{3}}T^{3}\right)$$

Let $V_{Q}$ denote the current base volume of $x^{i}$ system, and

(5) $$V_{Q}=\left(Q_{1},Q_{2},Q_{3}\right)=Q_{1}\cdot \left(Q_{2}\times Q_{3}\right).$$

Then,

(6) $$\sigma^{n}=\frac{1}{V_{Q}}T^{i}\frac{\partial n}{\partial x^{i}}$$

The key point to note here is that:

(7) $$\frac{\partial n}{\partial x^{i}}=Q_{i}\cdot n=n_{i}$$

Equation (6) can be derived as:

(8) $$\sigma^{n}=\frac{n_{i}}{V_{Q}}T^{i}$$

Then, for a small deformation case, the strain ε can be obtained as:

(9) $$\varepsilon =\frac{1}{2}\left(u_{i}⊗P^{i}+P^{i}⊗u_{i}\right)$$

The relationship between the base forces and the various stress tensors can be obtained according to the base force. 

As for the Cauchy stress, σ is:

(10) $$\sigma =\frac{1}{V_{Q}}T^{i}⊗Q_{i}$$

As for the Piola stress, τ is:

(11) $$\tau =\frac{1}{V_{P}}T^{i}⊗P_{i}$$

As for the Kirchhoff stress, Σ is:

(12) $$\Sigma =P_{i}⊗Q^{i}\frac{1}{V_{P}}T^{i}⊗P_{i}$$

The equilibrium equation is the balance of the stress, inertial force and volume forces of the structure. For static problems, the equilibrium equation can be expressed by the base force, as follows:

(13) $$\frac{\partial }{\partial x^{i}}T^{i}+\rho_{0}V_{P}f=0,$$

and the geometric equation can be obtained according to the displacement gradient:

(14) $$\varepsilon =\frac{1}{2}\left(u_{i}\cdot P_{j}+P_{i}\cdot u_{j}\right)P^{i}⊗P^{j}$$

Similarly, based on the base force, the physical equation is:

(15) $$T^{i}=\rho_{0}V_{P}\frac{\partial W}{\partial Q_{i}}$$

in which W is the strain energy of the element.

## 3. BFEM Model of Hexahedron Element

**Figure 3:**
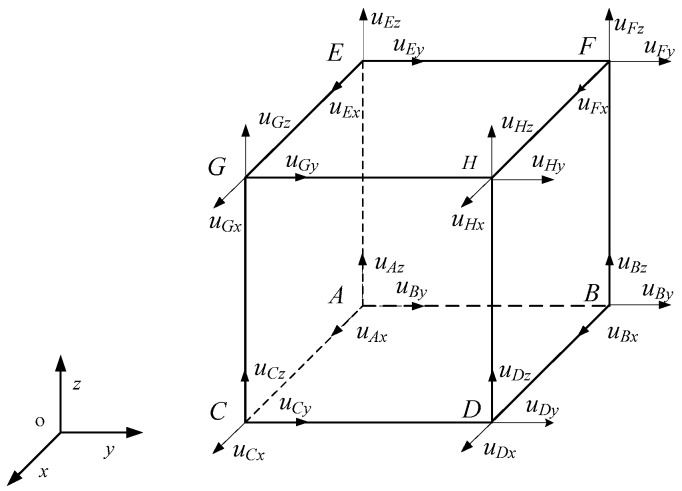
A hexahedron element.

Following the BFEM, a hexahedron element considering boundary conditions can be presented, as depicted in Figure 3. A,B,…,G are the vertices of the element, $u_{Ij}\left(I=A,B,\ldots G;j=x,y,z\right)$ denote the component of the displacement of the point I on the coordinate axes J, and α,β,γ… are the six areas of the model.

**Figure 4:**
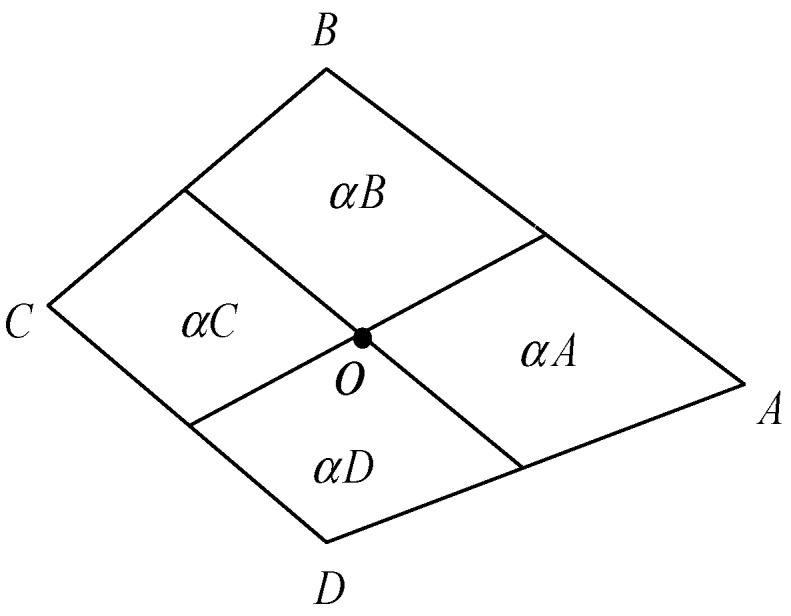
A typical face.

The hexahedron elements contact each other through the faces in the model. A relationship can be established between the displacements of the points and the displacements of the faces. Take any plane in the hexahedron as a typical face and let it be represented by α, as depicted in Figure 4. Connect the centroid point and the midpoint of the four sides; therefore, the quadrilateral is divided into four parts. Let $S_{\alpha }$ express the area of α and $S_{\alpha I}\left(I=A,B,C\ldots \right)$ denote the area of the separated part. 

It is hypothesized that in the process of deformation, the shape and line segments are always kept flat and straight, respectively. Hence, the deformation of the centroid can be obtained as:

(16) $$u_{\alpha }=\frac{1}{S_{\alpha }}\left(S_{\alpha A}u_{A}+S_{\alpha B}u_{B}+\cdot \cdot \cdot \right)$$

### 3.1. Strain Tensor 

Assume that the volume $V_{Q}$ of the hexahedron element is small complete and the actual strain ε can be replaced by the average strain $\bar{\varepsilon }$. In addition, the average strain $\bar{\varepsilon }$ can be obtained as:

(17) $$\bar{\varepsilon }=\frac{1}{V}\int_{V}\varepsilon dV$$

Then, by substituting Equation (9) into Equation (17), one can obtain:

(18) $$\bar{\varepsilon }=\frac{1}{2V}\int_{V}\left(u_{\alpha }⊗P^{\alpha }+P^{\alpha }⊗u_{\alpha }\right)\mathrm{dV}$$

According to Gauss theorem, Equation (18) can be replaced by:

(19) $$\bar{\varepsilon }=\frac{1}{2V}\left(u_{I}⊗m^{I}+m^{I}⊗u_{I}\right)$$

Equation (19) implies the summation rule, so $m^{I}$ is:

(20) $$m^{I}=S_{aI}n_{\alpha }+S_{\beta I}n_{\beta }+S_{\gamma I}n_{\gamma }+\ldots$$

The expressions of the hexahedron element are:

(21) $$\begin{array}{ll} m^{I} & =S_{\alpha I}n_{\alpha }+S_{\beta I}n_{\beta }+S_{\gamma I}n_{\gamma }\\ & =S_{\alpha I}\left(n_{\alpha x}e_{x}+n_{\alpha y}e_{y}+n_{\alpha z}e_{z}\right)+S_{\beta I}\left(n_{\beta x}e_{x}+n_{\beta y}e_{y}+n_{\beta z}e_{z}\right)\\ & \;+S_{\gamma I}\left(n_{\gamma x}e_{x}+n_{\gamma y}e_{y}+n_{\gamma z}e_{z}\right)\\ & =\left(S_{\alpha I}n_{\alpha x}+S_{\beta I}n_{\beta x}+S_{\gamma I}n_{\gamma x}\right)e_{x}+\left(S_{\alpha I}n_{\alpha y}+S_{\beta I}n_{\beta y}+S_{\gamma I}n_{\gamma y}\right)e_{y}\\ & \;+\left(S_{\alpha I}n_{\alpha z}+S_{\beta I}n_{\beta z}+S_{\gamma I}n_{\gamma z}\right)e_{z} \end{array}$$

Then, by substituting Equation (21) into Equation (19) and by letting x,y,z represent the Cartesian coordinate system, the following can be obtained:

(22) $$\begin{array}{ll} \bar{\varepsilon }= & \frac{1}{2V}\sum_{I=1}^{n}(2u_{Ix}m_{x}^{I}e_{x}⊗e_{x}+2u_{Iy}m_{y}^{I}e_{y}⊗e_{y}\\ & +2u_{Iz}m_{z}^{I}e_{z}⊗e_{z}+\left(u_{Ix}m_{y}^{I}+u_{Iy}m_{x}^{I}\right)e_{y}⊗e_{x}\\ & +\left(u_{Ix}m_{y}^{I}+u_{Iy}m_{x}^{I}\right)e_{x}⊗e_{y}+\left(u_{Ix}m_{z}^{I}+u_{Iz}m_{x}^{I}\right)e_{x}⊗e_{z}\\ & +\left(u_{Ix}m_{z}^{I}+u_{Iz}m_{x}^{I}\right)e_{z}⊗e_{x}+\left(u_{Iy}m_{z}^{I}+u_{Iz}m_{y}^{I}\right)e_{y}⊗e_{z}\\ & +\left(u_{Iy}m_{z}^{I}+u_{Iz}m_{y}^{I}\right)e_{z}⊗e_{y}) \end{array}$$

or

(23) ε¯x=1V∑I=1n(uIxmxI)  ε¯y=1V∑I=1n(uIymyI)ε¯z=1V∑I=1n(uIzmzI)  γ¯xz=1V∑I=1n(uIxmzI+uIzmxI)γ¯xy=1V∑I=1n(uIxmyI+uIymxI)  γ¯yz=1V∑I=1n(uIymzI+uIzmyI)

### 3.2. Stiffness Matrix

As the linear elastic material, the strain energy expression of the element can be obtained as:

(24) $$W_{D}=\frac{VE}{2\left(1+\nu \right)}\left[\frac{\nu }{1-2\nu }{\left(\bar{\varepsilon }:U\right)}^{2}+\bar{\varepsilon }:\bar{\varepsilon }\right]$$

In this formula, V denotes the hexahedron volume, E expresses the elastic modulus, ν expresses the Poisson’s ratio, and U denotes the unit tensor.

Then, by substituting Equation (19) into Equation (24), the strain energy can be obtained as:

(25) $$W_{D}=\frac{E}{4V\left(1+\nu \right)}\left[\frac{2\nu }{1-2\nu }{\left(u_{I}\times m^{I}\right)}^{2}+\left(u_{I}\cdot u_{J}\right)m^{IJ}+\left(u_{I}\cdot m^{J}\right)\left(u_{J}\cdot m^{I}\right)\right]$$

Based on Equation (25), the force applying to the node I(A,B,…G) on the element can be expressed as:

(26) $$f^{I}=\frac{\partial W_{D}}{\partial u^{I}}=K^{IJ}\cdot u_{J}$$

Then, the stiffness matrix K of the can be obtained as:

(27) $$K^{IJ}=\frac{E}{2V\left(1+\nu \right)}\left[\frac{2\nu }{1-2\nu }m^{I}⊗m^{J}+m^{IJ}U+m^{J}⊗m^{I}\right]$$

Here, $m^{I}=m_{i}^{I}e_{i}=m_{x}^{I}e_{x}+m_{y}^{I}e_{y}+m_{z}^{I}e_{z}$, and $m^{IJ}=m^{I}\cdot m^{J}$. 

By transforming Equation (27) into a Descartes coordinate system (x,y,z), the stiffness matrix K can be described to be:

(28) $$\begin{array}{ll} K^{IJ} & =\frac{E}{2V\left(1+\nu \right)}[e_{x}⊗e_{x}\left(\frac{2\nu }{1-2\nu }m_{x}^{I}m_{x}^{J}+m_{x}^{I}m_{x}^{J}+m_{y}^{I}m_{y}^{J}+m_{z}^{I}m_{z}^{J}+m_{x}^{I}m_{x}^{J}\right)\\ & +e_{x}⊗e_{y}\left(\frac{2\nu }{1-2\nu }m_{x}^{I}m_{y}^{J}+m_{y}^{I}m_{x}^{J}\right)+e_{y}⊗e_{x}\left(\frac{2\nu }{1-2\nu }m_{y}^{I}m_{x}^{J}+m_{x}^{I}m_{y}^{J}\right)\\ & +e_{x}⊗e_{z}\left(\frac{2\nu }{1-2\nu }m_{x}^{I}m_{z}^{J}+m_{z}^{I}m_{x}^{J}\right)+e_{z}⊗e_{x}\left(\frac{2\nu }{1-2\nu }m_{z}^{I}m_{x}^{J}+m_{x}^{I}m_{z}^{J}\right)\\ & +e_{y}⊗e_{z}\left(\frac{2\nu }{1-2\nu }m_{y}^{I}m_{z}^{J}+m_{z}^{I}m_{y}^{J}\right)+e_{z}⊗e_{y}\left(\frac{2\nu }{1-2\nu }m_{z}^{I}m_{y}^{J}+m_{y}^{I}m_{z}^{J}\right)\\ & +e_{y}⊗e_{y}\left(\frac{2\nu }{1-2\nu }m_{y}^{I}m_{y}^{J}+m_{x}^{I}m_{x}^{J}+m_{y}^{I}m_{y}^{J}+m_{z}^{I}m_{z}^{J}+m_{y}^{I}m_{y}^{J}\right)\\ & +e_{z}⊗e_{z}\left(\frac{2\nu }{1-2\nu }m_{z}^{I}m_{z}^{J}+m_{x}^{I}m_{x}^{J}+m_{y}^{I}m_{y}^{J}+m_{z}^{I}m_{z}^{J}+m_{z}^{I}m_{z}^{J}\right)] \end{array}$$

or

(29) $$K^{IJ}=\frac{E}{2V(1+\nu )}\left[\begin{array}{ccc} \frac{2-2\nu }{1-2\nu }m_{x}^{I}m_{x}^{J}+m_{y}^{I}m_{y}^{J}+m_{z}^{I}m_{z}^{J} & \frac{2\nu }{1-2\nu }m_{x}^{I}m_{y}^{J}+m_{y}^{I}m_{x}^{J} & \frac{2\nu }{1-2\nu }m_{x}^{I}m_{z}^{J}+m_{z}^{I}m_{x}^{J}\\ \frac{2\nu }{1-2\nu }m_{y}^{I}m_{x}^{J}+m_{x}^{I}m_{y}^{J} & \frac{2-2\nu }{1-2\nu }m_{y}^{I}m_{y}^{J}+m_{x}^{I}m_{x}^{J}+m_{z}^{I}m_{z}^{J} & \frac{2\nu }{1-2\nu }m_{y}^{I}m_{z}^{J}+m_{z}^{I}m_{y}^{J}\\ \frac{2\nu }{1-2\nu }m_{z}^{I}m_{x}^{J}+m_{x}^{I}m_{z}^{J} & \frac{2\nu }{1-2\nu }m_{z}^{I}m_{y}^{J}+m_{y}^{I}m_{z}^{J} & \frac{2-2\nu }{1-2\nu }m_{z}^{I}m_{z}^{J}+m_{x}^{I}m_{x}^{J}+m_{y}^{I}m_{y}^{J} \end{array}\right]$$

in which $m_{i}^{I}$ can be calculated:

(30) $$m_{x}^{I}=\frac{1}{4}S\left(n_{\alpha x}+n_{\beta x}+n_{\gamma x}\right)\;m_{y}^{I}=\frac{1}{4}S\left(n_{\alpha y}+n_{\beta y}+n_{\gamma y}\right)\;m_{z}^{I}=\frac{1}{4}S\left(n_{\alpha z}+n_{\beta z}+n_{\gamma z}\right)$$

where (I,J=1,2,3…,8) are the nodes of an element, and $n_{IJ}\left(I=\alpha ,\beta ,\gamma ;J=x,y,z\right)$ is the normal vector component of the air I about coordinate axes J.

## 4. Random Model of RAC

### 4.1. Aggregate Number

When considering the actual specimen of RAC, the aggregates are randomly distributed inside the test piece. Therefore, to obtain a more realistic mesostructure of RAC, the Fuller grading curve was adopted to calculate the amount of aggregate in the specimen. 

By assuming that the aggregate shape is spherical, the simple equation is as follows:

(31) $$P=100{\left(\frac{d}{D_{\max}}\right)}^{n}$$

where P denotes the cumulative distribution of the aggregate that was filtered through the diameter of sieve pore, d denotes the diameter of sieve pore, $D_{\max}$ denotes the maximum size of aggregate, n denotes the index, and, in this paper, n=0.5.

The volume of all aggregates in the grading interval $\left[d_{c},d_{c+1}\right]$ is defined as:

(32) $$V_{i}=0.5\times V\times \left(P_{ci}-P_{ci+1}\right)$$

where V is the specimen volume.

Then, the numbers of random spherical coarse aggregate particles with different diameters can be calculated:

(33) $$N_{i}=V_{i}/\left(\pi D_{i}^{3}/6\right)$$

where $D_{i}$ represents the sizes of the spherical coarse aggregate particles.

According to Equations (31)–(33), the amount of random spherical coarse aggregates with different radii can be calculated. The amount of recycled aggregate (RA) and natural aggregate (NA) with different replacement ratios are displayed in Table 1. 

### 4.2. Placing Algorithm

Following to Monte Carlo random method, three independent random numbers $R_{n},E_{n},F_{n}$ between 0 and 1 were generated to calculate the $x_{n},y_{n},z_{n}$ position of the aggregate particles. 

It should be noted that the placing algorithm should satisfy the following conditions: the named boundary condition and overlapping condition:

(1) The aggregate particles must be completely located in the specimen.
(2) The aggregate particles must not overlap with each other.
(3) The distance between centers of any two adjacent aggregate particles must be larger than that of 1.20 ($d_{a}+d_{b}$), where $d_{a}$ and $d_{b}$ are the radii of the two adjacent aggregates.

The placement process used can be summarized as follows:

Step 1: Generate three random numbers to get the particles coordinate.

Step 2: Check the boundary condition and the overlapping condition; if they do not meet the requirements, go back to Step 1.

Step 3: Place the aggregate into the specimen.

Step 4: Repeat the above steps for each aggregate.

**Figure 5:**
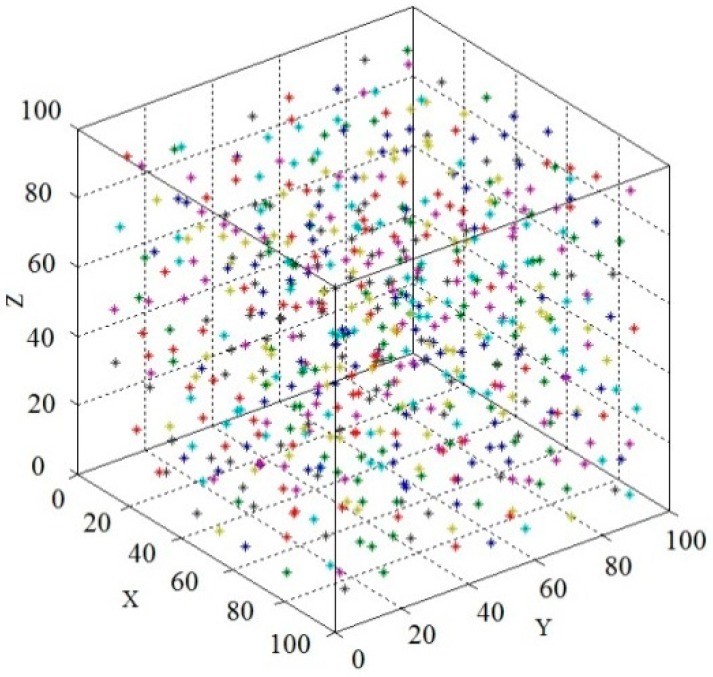
The aggregate coordinates scatter plot.

The obtained aggregate coordinates are depicted in Figure 5.

### 4.3. Numerical Model of RAC 

**Figure 6:**
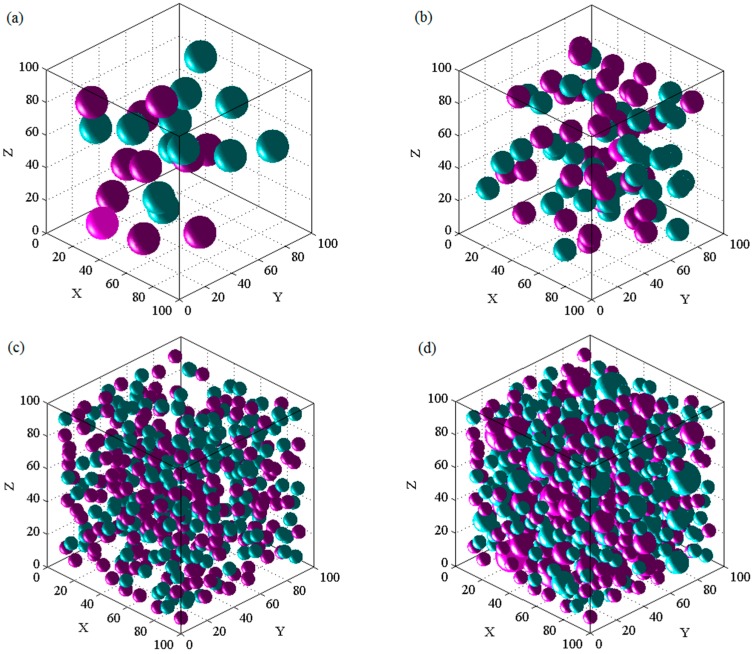
3D Random aggregate model of recycled aggregate concrete (RAC). (**a**) The radius is 8.75 mm; (**b**) the radius is 6.25 mm; and (**c**) the radius is 3.75 mm; (**d**) All aggregates.

The numerical models of RAC are displayed in Figure 6. Here, the dimension was 100 × 100 × 100 mm, and the replacement ratio was 50%. It should be mentioned that the dark blue aggregates and purple aggregates represent the natural and recycled aggregates, respectively. 

**Figure 7:**
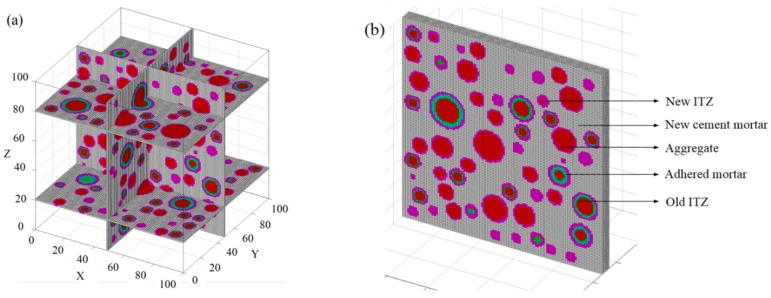
Slices and five-phase in the model. (**a**) Four slices in the model. (**b**) Five-phase system.

As can be observed in Figure 6, the aggregates had a good distribution and did not overlap with one another. In addition, four slices were extracted from the model to verify the accuracy of the placing algorithm. Here, the mesh size was 0.8 × 0.8 mm, as detailed in Figure 7a. Meanwhile, the five-phase system is also indicated in Figure 7b.

### 4.4. Mechanical Parameters

It is well accepted that the concrete is regarded as a quasi-brittle material, and lots of damage constitutive models have been presented [26,27,28]. In this work, the failure mechanical behavior of the five-phase system was described by a bilinear failure model [7].

According to the experiment results of [29,30,31,32] and numerical results of [7,23,33], it has been accepted that the mechanical properties of ITZs are weaker than those of the corresponding cement mortars. In addition, their elastic modulus is randomly distributed as 0.5–0.85 times that of cement mortar. Therefore, for this paper, the elastic modulus of the ITZs was selected as 0.55 times of the corresponding cement mortars, as noted in Table 2.

## 5. Simulation of Uniaxial Compressive Test 

For this section, several RAC models were applied to the uniaxial compression loading. All nodes of the bottom elements and the nodes of the mid-bottom elements were restrained in the vertical direction and horizontal direction, respectively. In addition, the displacement loading was applied to the nodes of the top elements at 0.005 mm/step. These models were used to explore the influences of recycled aggregate substitution and the adhered mortar percent on the elastic modulus, the compressive strength, and the crack pattern. Therefore, (1) five different substitutions of recycled aggregate (0%, 15%, 30%, 50%, and 100%) were established for these models, and the adhered mortar was chosen as 40%; (2) another RAC model with one recycled aggregate was established, and six levels of adhered mortar percentage (0%, 5%, 10%, 30%, 40%, and 50%) of the recycled aggregate were designed; and (3) the RAC model with one aggregate was cut off to display the occurrence and development of cracks inside the specimen, with the percentage of the adhered mortar being 40%.

### 5.1. Effect of Aggregate Substitution 

It is well known that the substitution of recycled aggregate is a major factor that affects mechanical properties. When increasing the substitution portion of natural aggregates by the recycled aggregate, both the elastic modulus and compressive strength decrease [28]. The obtained data are shown in Table 3.

As can be seen from Table 3, when the substitution was less than 30%, despite the reduction in the elastic modulus and compressive strength, only slight differences were obtained. However, when the substitution was increased from 0% to 100%, both the modulus of elasticity and the compressive strength showed reductions of up to 15.6% and 10.8%, respectively. 

These phenomena can be attributed to the increasing substitution of recycled aggregate, which resulted in an increase in the mortar adhering to recycled aggregate and the old ITZ between them. These two increased phases were considered to be the weak phase in the specimen and had lower mechanical properties. Therefore, as the substitution increased, the elastic modulus of the specimen decreased. These observed data coincide with other results in the literature [9,34,35,36,37].

### 5.2. Effect of Adhered Mortar Percentage 

Due to how waste concrete is dealt with, it is inevitable that some adhered mortar will remain around the surface of the aggregate. The physical property of the recycled aggregate depends on the percent and property of the adhering mortar. Previous studies have shown that the adhered mortar is a major factor that weakens the mechanical properties of RAC [36,38,39]. 

In this section, it should be mentioned that only one recycled aggregate was placed to test the effect of adhered mortar on the mechanical properties of RAC. This design avoided the influences of aggregate grading and aggregate distribution on its properties. Therefore, it was meaningless to use this model to investigate the values of the mechanical properties of RAC. Consequently, only the relative values of compressive strength and modulus of elasticity are given, as listed in Table 4.

As listed in Table 4, with the increasing percentage of adhered mortar, both the modulus of elasticity and compressive strength decreased. This was the same effect as that of the substitution—when the percent of the adhering mortar was less than 10%, the value of the mechanical properties showed a slight reduction. As the percentage of the adhered mortar increased from 0% to 50%, the compressive strength and elastic modulus decreased by 12% and 18%, respectively. These results can be attributed to the lower mechanical properties of the adhered mortar than that of the new cement mortar and aggregate. Consequently, this weak phase weakened the compressive strength and elastic modulus of RAC.

### 5.3. Failure Mechanism 

As follows experimental and numerical works [7,9,40,41,42], the first cracks always appeared in old and new ITZs and then propagated into the old and new cement mortar, resulting in several continuous cracks.

**Figure 8:**
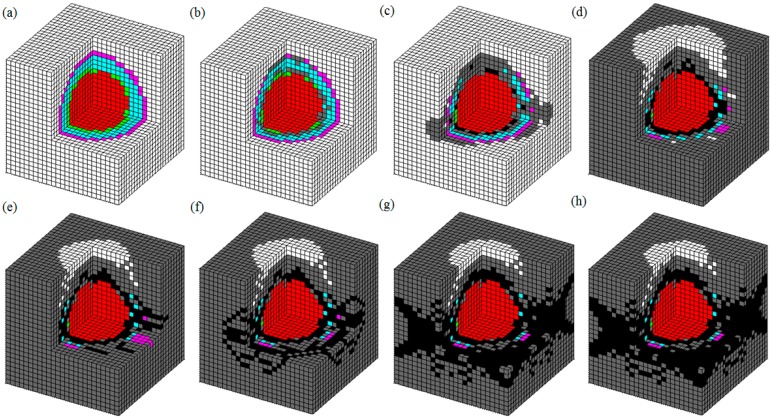
Failure pattern of RAC. (**a**) Initial model; (**b**) Elastic deformation; (**c**) First crack; (**d**~**f**) Crack propagation; (**g**,**h**) Continuous cracks.

Due to the limitation of the technology, it was difficult to view the failure pattern during the macroscopic experiment loading, especially that of the damage process of internal materials. To explore the damage mechanism of RAC, the crack pattern of a 3D model with one aggregate was created, as illustrated in Figure 8. 

From Figure 8, one can see that the first crack was formed in the old ITZ zones and then appeared in the new ITZ and adhered mortar. As the loading increased, several isolated cracks appeared in the cement mortar, and approximately two-to-three continuous cracks were formed in the specimen. As displayed in Figure 8g,h, the continuous cracks were inclined in the direction of 45°, and the materials in the middle of the specimen appeared to fall off. In addition, due to the restraining action on the bottom and top of the specimen, there was no failure crack in these zones. These phenomena can be attributed to the inferior properties of the ITZ and adhering mortar materials to other media. In a five-phase system, the mechanical properties of ITZs are the weakest, so cracks always appeared in these areas. Then, the stress of the adhered mortar adjacent to the damaged ITZs increased, and several isolated cracks were formed. When increasing the loading, a plurality of cracks was formed in the new cement mortar near the damaged ITZ and the adhered mortar. Finally, two-to-three continuous cracks were distributed in the specimen. These results showed a good agreement with other numerical and experimental data [13,22,40,41,42,43]. 

## 6. Conclusions

This paper developed a new finite element method named the 3D base force element method (BFEM) that can be applied to analyze the damage behavior of materials. According to the 3D BFEM, the stiffness matrix and node displacement of a hexahedron element were derived. A 3D model of recycled aggregate concrete (RAC) with sphere aggregates was established. These models were applied to the uniaxial compression loading that was controlled by displacement loading. The effects of the replacement ratio of the recycled aggregate and the percentage of adhering mortar on the elastic modulus and compressive strength were explored. Additionally, the failure pattern was also displayed. According to the research and data described in this work, several conclusions can be reached as follows.

(1) The 3D BFEM can be used to explore the failure mechanism of heterogeneous materials. The stiffness matrix and the node displacement of a hexahedron element can be derived as an explicit expression and without the use of Gaussian integration.

(2) The 3D placing algorithm of the RAC numerical model is feasible to characterize the random structure of an aggregate in a specimen. The mesostructure and the mechanical behavior of RAC can be characterized by this numerical model. 

(3) The replacement ratio of recycled aggregate has a major influence on mechanical properties. When increasing substitution, both the elastic modulus and compressive strength reduce. The substitution should be controlled below 30% in civil engineering.

(4) The percentage of the adhering mortar around the surface of the recycled aggregate has a negative influence on mechanical properties. The modulus of elastic and compressive strength decrease as the percentage increases. The waste concrete should be treated in a reasonable and inexpensive manner to advance the quality and performance of the recycled aggregate.

(5) The weak mechanical properties of an old ITZ and adhered mortar are major factors that cause the mechanical property degradation of RAC than that of NAC. In addition, these two phases have a significant influence on the failure mechanism of RAC.

## Figures and Tables

**Table 1:** The amount of the aggregate.

Replacement Ratio	Aggregate Radius (mm)					
	7.5		12.5		17.5	
	RA	NA	RA	NA	RA	NA
0%	0	468	0	77	0	23
15%	70	398	12	65	3	20
30%	140	328	23	54	7	16
50%	234	234	38	39	11	12
100%	468	0	77	0	23	0

**Table 2:** Mechanical properties of the five-phase system.

Mechanical Properties	Five Phases				
	Natural Aggregate	Old ITZ	Adhered Mortar	New ITZ	New Cement Mortar
Elastic modulus/GPa	75	13.75	25	16.50	30
Poisson’s ratio	0.16	0.20	0.22	0.20	0.22
Tensile strength/MPa	10.0	2.0	2.5	2.0	3.0

**Table 3:** Effect of substitution on the mechanical properties of RAC.

Mechanical Properties	Replacement Ratio				
	0%	15%	30%	50%	100%
Elastic modulus/GPa	25.56	24.29	22.36	22.06	21.34
Compressive stress/MPa	28.09	26.08	25.23	25.19	25.06

**Table 4:** Effect of the percent of adhering mortar on the mechanical properties of RAC.

Mechanical Properties		Percentage of Adhered Mortar				
	0%	5%	10%	30%	40%	50%
Elastic modulus/GPa	1	0.96	0.93	0.86	0.84	0.82
Compressive stress/MPa	1	0.97	0.95	0.92	0.91	0.88

Note: 0% corresponds to natural aggregate concrete.
